# Overexpression of Osteopontin, αvβ3 and Pim-1 Associated with Prognostically Important Clinicopathologic Variables in Non-Small Cell Lung Cancer

**DOI:** 10.1371/journal.pone.0048575

**Published:** 2012-10-31

**Authors:** Yi Jin, Da-yue Tong, Jian-ning Chen, Zhi-ying Feng, Jian-yong Yang, Chun-kui Shao, Jia-ping Li

**Affiliations:** 1 Department of Pathology, the Third Affiliated Hospital, Sun Yat-Sen University, Guangzhou, China; 2 Department of Forensic medicine, ZhongShan Medical School, Sun Yat-Sen University, Guangzhou, China; 3 Department of Interventional Oncology, the First Affiliated Hospital, Sun Yat-Sen University, Guangzhou, China; Ospedale Pediatrico Bambino Gesù, Italy

## Abstract

In this study, we examined the expression of osteopontin (OPN), αvβ3 and Pim-1 in non-small cell lung cancer (NSCLC) and investigated the potential clinical implications of their expression patterns in NSCLC. Immunohistochemical assays were used to examine the protein expression of OPN, αvβ3 and Pim-1 in 208 NSCLC samples and their adjacent normal lung tissue specimens. Statistical analyses were performed to evaluate the relationships between OPN, αvβ3 and Pim-1 expression patterns, and their association with the clinical-pathological parameters of NSCLC patients. In NSCLC tissues, the positive rates of OPN, αvβ3 and Pim-1 expression were 67.8% (141/208), 76.0% (158/208) and 58.7% (122/208), respectively. However, in the adjacent normal lung tissues, the positive rates of OPN, αvβ3 and Pim-1 were 20.2% (42/208), 24.0% (50/208) and 14.9% (31/208), respectively. The differences in the positive expression rates of OPN, αvβ3 and Pim-1 between NSCLCs and the adjacent normal lung tissues were all significant (*P*<0.01). Additionally, the positive expression of OPN, αvβ3 and Pim-1 in NSCLCs was associated with an increase in pathological grade, lymph node metastasis and advanced clinical stage (all *P*<0.01). Furthermore, associations between the expression of OPN and αvβ3, OPN and Pim-1, and αvβ3 and Pim-1 were also observed in our NSCLC cohort (all *P*<0.01). The OPN, αvβ3 and Pim-1 proteins are frequently overexpressed in NSCLC and are associated with some clinicopathologic variables that are of known prognostic importance in NSCLC, suggesting that they may play an important role in the development and/or progression of NSCLC.

## Introduction

Primary bronchogenic carcinomas of the lung have the highest mortality rate of any malignant tumor in the world, and non-small cell lung cancer (NSCLC) accounts for 85% of primary lung cancers. Most NSCLC patients are clinically diagnosed at more advanced stages and have a very low 5-year survival rate [1]. Thus, early diagnosis and early treatment are particularly important for improving the survival rate. It is commonly accepted that various pathogenic factors are involved in the evolution of NSCLC.

Osteopontin (OPN) is a multifunctional secreted phosphorylated glycoprotein that promotes cellular chemotaxis, adhesion and migration. These processes mediate the invasion and metastasis of tumor cells and are associated with the occurrence, development, metastasis and prognosis of a variety of cancer types [2], [3], [4]. Recent studies have indicated that OPN is involved in NSCLC progression and metastasis through its interaction with the αvβ3 (alphavbeta3) integrin receptor, and OPN overexpression in NSCLC is associated with the pathological stage of the tumor, which is one of the predictor of poor prognosis [5]. The heparin-binding αvβ3 integrin mediates cell-cell and cell-matrix adhesion, regulates intracellular signaling pathways and induces the activation of protein-dissolving enzymes, thereby contributing to extracellular matrix and basement cell membrane degradation and promoting the invasion and migration of tumor cells [6]. Previous studies found that αvβ3 is detected in a variety of tumor types and is closely associated with both tumor development and the degree of tumor malignancy [7]. Other factors that are closely related with genes of cell cycle regulation and proliferation, such as Pim-1 (pim-1 oncogene), are also involved in tumorigenesis [8]. Reportedly, OPN acts through αvβ3 integrin, which in turn activates the FAK, PI3K, Akt, ERK, NF-κB and Pim-1 pathways, thus contributing to the migration of lung cancer cells [8]. Concurrently, these results suggest that OPN mediates migration in human lung cancer cells via the αvβ3 integrin, FAK, PI3K, Akt, ERK, NF-κB and Pim-1 signaling pathways. Therefore, we chose to further investigate OPN, αvβ3 integrin and Pim-1, three components of these signaling pathways, in this study. To date, however, the expression dynamics of OPN, αvβ3 and Pim-1 in NSCLCs and their potential biological roles in the tumorigenesis of NSCLC have not been fully elucidated.

In this study, immunohistochemical assays were used to determine the expression rates of OPN, αvβ3 and Pim-1 in 208 NSCLC samples and their adjacent normal lung tissue specimens. Additionally, the potential associations between the expression patterns of the three markers and their associations with the clinico-pathological features of NSCLC patients were evaluated.

## Materials and Methods

### Ethics Statement

This study was approved by the Clinical Research Ethics Committee of the Third Affiliated Hospital, Sun Yat-sen University. Written informed consent was received from each patient, and the ethical guidelines as detailed in the Declaration of Helsinki were followed.

### Subjects

In this study, specimens were obtained from the archived paraffin-embedded tissue sections of 208 consecutive NSCLC cases diagnosed at the Third Affiliated Hospital, Sun Yat-sen University, Guangzhou, China, from January 1, 2008 to December 31, 2010. Adjacent normal lung tissues from each case were used as controls. In this NSCLC cohort, 147 patients were male and 61 patients were female, with a median age of 60 years (range 30–82 years). According to the World Health Organization criteria of lung cancer published in 2004 [9], the histology of our NSCLC cohort was classified as follows: adenocarcinoma: 110 cases, squamous cell carcinoma: 61 cases, and other types: 37 cases; well differentiated carcinoma: 65 cases, moderately differentiated carcinoma: 70 cases, and poorly differentiated carcinoma: 73 cases. According to the TNM system from the International Association for the Study of Lung Cancer [10], 87 cases were classified as stage I, 59 cases were stage II, 42 cases were stage III and 20 cases were stage IV.

### Immunohistochemistry

Immunohistochemical techniques that use streptavidin-peroxidase (S-P) were employed for OPN, αvβ3 and Pim-1 detection. Ready-to-use mouse anti-human monoclonal antibodies against OPN, αvβ3 and Pim-1, and S-P kits were purchased from Maxim biological and technical company, Fuzhou, China. All sections were routinely deparaffinized and rehydrated, rinsed in phosphate-buffered saline (PBS, pH 7.4) and treated for antigen retrieval. Sections were treated in EDTA buffer (pH 8.0) in an autoclave sterilizer. After cooling at room temperature for 20 min, the sections were rinsed in PBS then immersed in 3% H_2_O_2_ for 15 min to block endogenous enzyme activity. After rinsing with PBS, the sections were incubated with normal goat serum at 37°C for 15 min to block nonspecific antibody binding. Following incubation with the primary antibodies (OPN, αvβ3 and Pim-1 monoclonal antibodies), the sections were rinsed in PBS, incubated with biotinylated secondary antibodies and rinsed with PBS again. After the sections were incubated with streptavidin-HRP and rinsed with PBS, the sections were visualized using 3,3′-diaminobenzidine and counterstained with hematoxylin. Finally, the sections were dehydrated, transparented, covered with coverslips and sealed with neutral gum. PBS without primary antibody was used as the negative control.

The results of the immunohistochemical assays were assessed by three of the authors (Jin Y, Chen JN and Shao CK). Positive OPN and Pim-1 expression was observed in the cytoplasm as a brown-yellow color, while αvβ3 was located in both the cytoplasm and the cell membrane as a brown-yellow color. Only cells with a clear cytoplasmic and/or membranous staining were regarded as positive. For each case, a total of 10 randomly selected high power fields (400×) were evaluated and the percentage of cells that showed positive staining was quantified. To minimize interindividual interpretation differences, the mean score of the three observers was used for analysis. A tumor or normal tissue in which greater than 10% of cells were positively stained was classified as positive [11], [12], [13]. A tumor or normal tissue with less than 10% positively stained cells was classified as negative.

### Statistical analysis

The expressions of the three markers (OPN, αvβ3 and Pim-1) were presented in the present study as dichotomy (positive/negative), and were analyzed as dichotomous variables. Chi-squared tests were used to compare the expression rates of OPN, αvβ3 and Pim-1 in NSCLCs and their adjacent normal lung tissues, as well as the associations between the expression of OPN, αvβ3 and Pim-1 and the clinico-pathological parameters of NSCLC patients. The associations between two variables were also evaluated by the chi-squared tests. Differences were considered to be statistically significant at a *p*-value of less than 0.05. All of the *p*-values presented in this study are two-sided. The data were analyzed with computer-aided SPSS13.0 statistical software.

## Results

### Expression of OPN and its association with the clinico-pathological parameters of NSCLC patients

**Figure 1:**
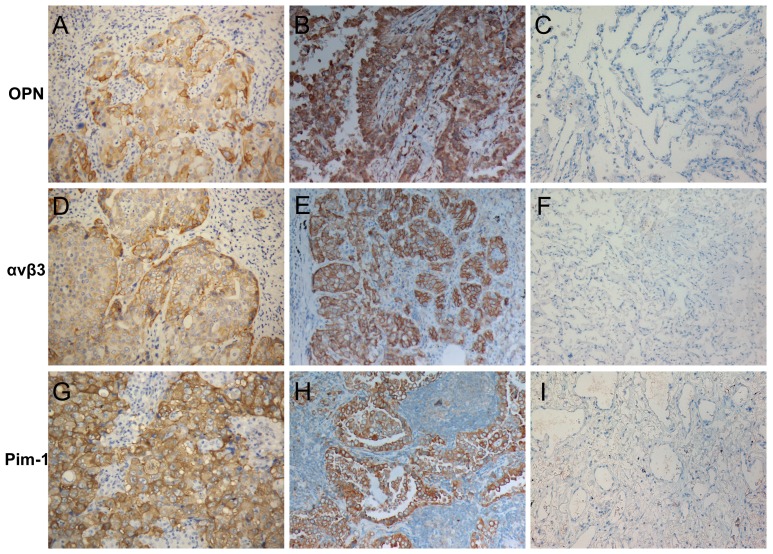
Expression of OPN, αvβ3 and Pim-1 in NSCLCs and adjacent normal lung tissues. OPN is expressed in the cytoplasm and appears as either yellow or brown-yellow. Positive expression of OPN was detected in a squamous carcinoma (A) and an adenocarcinoma (B) of the lung but not in the adjacent normal lung tissue (C). αvβ3 is expressed in cell membrane and cytoplasm and appears as either yellow or brown-yellow. αvβ3 is positive in a different squamous carcinoma (D) and an adenocarcinoma (E) of the lung but not in the adjacent normal lung tissue (F). The expression of Pim-1 is predominantly cytoplasmic. Pim-1 is positive in a squamous carcinoma (G) and an adenocarcinoma (H) of the lung but not in the adjacent normal lung tissue (I) (SP 20×10).

The expression of OPN in NSCLC tissues was predominantly cytoplasmic (Figure 1A-C). In our study, 141 of 208 (67.8%) NSCLC cases showed positive expression of OPN, while the positive rate of OPN expression in adjacent normal lung tissues was 20.2% (42/208). The difference between the expression of OPN in NSCLC tissues and the adjacent normal lung tissues was significant (*P*<0.01, Table 1). Further analyses demonstrated that the expression of OPN was significantly associated with the tumor differentiation degree, lymph node metastasis and clinical staging in NSCLC patients (*P*<0.01). The association between the expression of OPN and distant metastasis in NSCLC patients was of borderline statistically significance (*P* = 0.05). However, OPN expression was not correlated with other studied clinico-pathological parameters (*P*>0.05, Table 2).

**Table 1:** The expressions of OPN, αvβ3 and Pim-1 in NSCLCs and adjacent normal lung tissues.

Proteins	N	NSCLC	Adjacent normal lung tissue	?^2^ *	*P* *
		n (%)	n (%)		
OPN	208	141 (67.8)	42 (20.2)	95.62	<0.01
αvβ3	208	158 (76.0)	50 (24.0)	112.15	<0.01
Pim-1	208	122 (58.7)	31 (14.9)	85.61	<0.01

* The comparison is between the malignant and normal tissue for each marker.

**Table 2:** Relationship between expressions of OPN, αvβ3 and Pim-1 and clinico-pathological parameters of NSCLC.

Parameter	n	OPN (%)	*P*	αvβ3 (%)	*P*	Pim-1 (%)	*P*
Sex							
Male	147	102 (69.4)	0.44	113 (76.9)	0.63	85 (57.8)	0.71
Female	61	39 (63.9)		45 (73.8)		37 (60.7)	
Age (years)							
<60	93	62 (66.7)	0.76	71 (76.3)	0.91	55 (59.1)	0.90
≥60	115	79 (68.7)		87 (75.7)		67 (58.3)	
Tumor size							
<3	79	53 (67.1)	0.87	59 (74.7)	0.74	44 (55.7)	0.50
≥3	129	88 (68.2)		99 (76.7)		78 (60.5)	
Lymphnode metastasis*							
N0	140	82 (58.6)	<0.01	95 (67.9)	<0.01	71 (50.7)	0.01
N1	32	27 (84.4)		28 (87.5)		23 (71.9)	
N2	21	18 (85.7)		20 (95.2)		16 (76.2)	
N3	15	14 (93.3)		15 (100)		12 (80)	
Distant metastasis*							
M0	188	124 (66.0)	0.05	140 (74.5)	0.17	106 (56.4)	0.04
M1	20	17 (85)		18 (90)		16 (80)	
Differentiation							
Well	65	37 (56.9)	<0.01	43 (66.2)	<0.01	27 (41.5)	<0.01
Moderate	70	41 (58.6)		48 (68.6)		31 (44.3)	
Low	73	63 (86.3)		67 (91.8)		64 (87.7)	
Pathology typing							
Squamous carcinoma	61	39 (63.9)	0.74	47 (77.0)	0.89	35 (57.4)	0.97
Adenocarcinoma	110	76 (69.1)		84 (76.4)		65 (59.1)	
Other types	37	26 (70.3)		27 (73.0)		22 (59.5)	
Tumor location							
Peripheral	82	54 (65.9)	0.63	61 (74.4)	0.67	46 (56.1)	0.55
Central	126	87 (69.0)		97 (77.0)		76 (60.3)	
TNM stage*							
I	87	51 (58.6)	<0.01	60 (69.0)	<0.01	43 (49.4)	<0.01
II	59	35 (59.3)		41 (69.5)		30 (50.9)	
III	42	38 (90.5)		39 (92.9)		33 (78.6)	
IV	20	17 (85)		18 (90)		16 (80)	

* According to the TNM system from the International Association for the Study of Lung Cancer [10]

### Expression of αvβ3 and its association with the clinico-pathological parameters of NSCLC patients

In NSCLCs, we observed that the αvβ3 protein was expressed mostly in the cell membrane or cytoplasm (Figure 1D-F). The positive rate of αvβ3 expression in NSCLCs was 76.0% (158/208), which was significantly higher than in adjacent normal lung tissues (24.0%, 50/208) (*P*<0.01, Table 1). In addition, the expression of αvβ3 was also positively associated with histopathological differentiation, lymph node metastatic status and clinical stage (*P*<0.01, Table 2).

### Expression of Pim-1 and its association with the clinico-pathological parameters of NSCLC patients

Pim-1 expression was observed primarily in cytoplasm (Figure 1G-I). In our NSCLC cohort, 122 of the 208 (58.7%) cases showed positive expression of Pim-1, while the positive rate of Pim-1 expression in normal lung tissues was 14.9% (31/208). The difference between Pim-1 expression in NSCLC tissues and normal lung tissues was significant (*P*<0.01, Table 1). Additionally, the expression of Pim-1 was significantly correlated to the differentiation degree, lymph node metastasis, distant metastasis and clinical staging in NSCLC tissues (*P*<0.01), but no correlations were observed between the expression of Pim-1 and other studied clinico-pathological parameters (*P*>0.05, Table 2).

### Associations between the protein expression patterns of OPN, αvβ3 and Pim-1 in NSCLCs

In our study, the potential associations between the protein expression patterns of OPN, αvβ3 and Pim-1 in NSCLCs were further evaluated. 89.4% (126/141) of tumors positive on OPN were also αvβ3-positive, and 52.2% (35/67) OPN negative tumors were αvβ3 negative. The association between the expression of OPN and αvβ3 was statistically significant (*P*<0.01, Table 3). Besides, 72.3% (102/141) of tumors positive on OPN were positive for Pim-1, and 70.1% (47/67) of tumors negative on OPN were also Pim-1 negative. The association between the expression of OPN and Pim-1 was also statistically significant (*P*<0.01, Table 4). Furthermore, 66.5% (105/158) of tumors positive for αvβ3 were positive for Pim-1, and 66% (33/50) of tumors negative for αvβ3 were also Pim-1 negative. An association between the expression of αvβ3 and Pim-1 was also observed (*P*<0.01, Table 5).

**Table 3:** Relativity of protein expressions of OPN and αvβ3 in NSCLC.

OPN	αvβ3		total
	positive (%)	negative (%)	
positive	126 (89.4)	15 (10.6)	141
negative	32 (47.8)	35 (52.2)	67
total	158	50	208

The association between the expression of OPN and αvβ3 in NSCLC was statistically significant (χ^2^ = 42.84, *P*<0.01).

**Table 4:** Relativity of protein expressions of OPN and Pim-1 in NSCLC.

OPN	Pim-1		total
	positive (%)	negative (%)	
positive	102 (72.3)	39 (27.7)	141
negative	20 (29.9)	47 (70.1)	67
total	122	86	208

The association between the expression of OPN and Pim-1 in NSCLC was statistically significant (χ^2^ = 33.65, *P*<0.01).

**Table 5:** Relativity of protein expressions of αvβ3 and Pim-1 in NSCLC.

αvβ3	Pim-1		total
	positive (%)	negative (%)	
positive	105 (66.5)	53 (33.5)	158
negative	17 (34)	33 (66)	50
total	122	86	208

The association between the expression of αvβ3 and Pim-1 in NSCLC was statistically significant (χ^2^ = 16.42, *P*<0.01).

## Discussion

It is well-established that the malignant transformation of cells requires changes of gene phenotype and angiogenesis. OPN is considered to be the most important factor for malignant transformation. OPN, also well known as a transformation-related protein phosphatase, is an extracellular matrix secreted phosphorylated glycoprotein that was originally found by Senger and colleagues [14] in epithelial cells that had undergone malignant transformation. In recent years, additional studies have confirmed that overexpression of OPN can promote tumor growth, invasion and/or metastasis [3].

Previously, it was reported that the expression of OPN was observed in 68.8% in NSCLC cases [15]. Other groups reported that OPN expression was associated with tumor growth, tumor staging and lymph node invasion of patients with NSCLC [16]. In our present study, the expression rate of OPN protein in NSCLCs was determined to be 67.8%, while only 20.2% of normal lung tissues expressed OPN protein, suggesting that OPN expression may provide a selective advantage for the development of NSCLC. Further statistical analyses showed that OPN expression correlated closely with the differentiation degree of NSCLC, lymph node metastasis and clinical staging but that it was independent of other clinico-pathological parameters of NSCLCs. Additionally, OPN expression was more frequently observed in poorly differentiated cancers, tumors with lymph node metastasis and/or tumors of advanced clinical stage (III/IV). A current study suggests that OPN can produce a marked effect by binding to receptors on endothelial cell cell membranes. Following OPN binding to receptors such as the αvβ3 integrins, it can directly stimulate the differentiation and proliferation of lung cancer cells, and may regulate the genesis and migration of lung cancer cells, increase vascular permeability, alter the extracellular matrix, induce angiogenesis, activate intracellular signaling pathways and promote the growth of NSCLC [17]. These data suggest that the increased expression of OPN may facilitate the development and/or progression of NSCLC, and it is possible that OPN could be used as either a therapeutic target or a biomarker for NSCLC in the future.

High OPN expression in NSCLC may result from its regulation by transcription factors such as αvβ3 and Pim-1. Previous studies have demonstrated that αvβ3 regulates the expression of Pim-1 and VEGF, and mediates tumor angiogenesis through the PI3/AKT signaling pathways. Consequently, they also promote the expression of OPN [18], [19]. Recently, it has been reported that the overexpression of αvβ3 integrin is associated with an aggressive phenotype in several solid tumor types [20], [21]. In this study, we found that the expression rate of αvβ3 was 76.0% in NSCLC tissues, which was significantly higher than in normal lung tissues (24.0%), suggesting that αvβ3 may be involved in the development of NSCLC. In addition, statistical analyses showed that the protein expression of αvβ3 was positively associated with the differentiation degree of NSCLC, lymph node metastasis and clinical staging. These results further suggest that the expression of αvβ3 may be involved in the development of NSCLC, as well as the degree of malignancy, invasion and metastasis of NSCLC. A possible molecular mechanism that may explain how αvβ3 promotes the development of NSCLC is the role that αvβ3 integrin plays in promoting angiogenesis and the cell-matrix adhesion [22]. Thus, it is possible that αvβ3 is used as an important indicator to evaluate the degree of malignant invasion in NSCLC. It is also possible that αvβ3 is used as a surrogate marker in the diagnosis of NSCLC and a predictor of the therapeutic response following treatment with the anti-αvβ3 antibody in NSCLC patients. However, further research is required to determine their accuracy.

Pim-1, a potential oncogene, is located on chromosome 6p21.2 and encodes a serine/threonine kinase [23]. Recent studies have confirmed that it is highly expressed in a subset of malignant tumors [24], [25]. Pim-1 regulates the proliferation, differentiation and apoptosis of lung cancer cells [26]. In our present study, Pim-1 expression was detected in the majority of NSCLCs, while Pim-1 expression was observed in a significantly lower percentage of normal lung tissues. This result is consistent with the findings reported by Zhang et al. [27] and He et al. [28]. It has been documented that Pim-1 can promote G2/M, and activate cell growth, differentiation and proliferation [8]. We also found that Pim-1 protein expression correlates with poorly differentiated NSCLC tissues, advanced clinical stage (III/IV) and lymph node and distant metastasis at significantly higher rates than early clinical stage (I/II) NSCLC cases and no lymph node and/or distant metastasis. Based on these collective results, we propose that Pim-1 may also be involved in the tumorigenesis and/or progression of NSCLC.

In this study, statistical analysis showed that there was a strong positive-positive association between the expression of OPN and αvβ3. It was suggested that OPN acts through αvβ3 integrin, which in turn activates the FAK signaling pathways and further contributes to the overexpression of αvβ3 [29]. Other groups also found that OPN is involved in both the tumor growth and angiogenesis of lung cancer by up-regulating vascular endothelial cell migration and proliferation through its interaction with αvβ3 integrin [30]. Upregulation of OPN specifically activates the activity of the αvβ3 integrin receptor, which may accelerate tumor angiogenesis and induce the activation of a variety of kinases such as Pim-1, PI3K and AKT [18], [19]. Therefore, higher OPN expression levels can elevate the expression of αvβ3 and Pim-1 by activating additional signaling pathways. In our present study, the expression of OPN, αvβ3 and Pim-1 in NSCLC were associated with each other. Therefore, our results, together with findings by other groups, suggest that OPN plays a crucial role for tumor growth and/or progression of human lung cancer by interacting with αvβ3 integrin and activating Pim-1 through additional signaling pathways [18], [19], [23], [29], thus resulting in the overexpression of OPN, αvβ3 and Pim-1.

In conclusion, the present study is the first research evaluating the relationship among osteopontin, αvβ3 and Pim-1, and their association with the clinical-pathological parameters of NSCLC. Although all analyses were univariate, the present study showed that the OPN, αvβ3 and Pim-1 proteins are frequently overexpressed in NSCLC and are associated with some clinicopathologic variables which are of known prognostic importance in NSCLC, suggesting that they may play an important role in the development and/or progression of NSCLC. Targeting the interaction between OPN, αvβ3 and/or Pim-1 may be effective in the future development of anti-angiogenic therapeutic agents for NSCLC patients.
